# Outcomes of Primary Ligation of Patent Ductus Arteriosus Compared With Secondary Ligation After Pharmacologic Failure in Very-Low-Birth-Weight Infants

**DOI:** 10.1007/s00246-013-0854-6

**Published:** 2013-12-27

**Authors:** Young-Ah Youn, Cheong-Jun Moon, So-Young Kim, Jae Young Lee, In-Kyung Sung

**Affiliations:** 1 Department of Pediatrics, Catholic Medical College, Seoul St. Mary’s Hospital, Seoul, Republic of Korea; 2 Department of Pediatrics, Catholic Medical College, Yeouido St. Mary’s Hospital, Seoul, Republic of Korea

**Keywords:** Surgical ligation, Morbidity, Hemodynamically significant ductus arteriosus

## Abstract

This study aimed to determine whether primary surgical closure of patent ductus arteriosus (PDA) is a risk factor for morbidity and mortality compared with secondary surgical ligation. The study enrolled 178 very-low-birth-weight infants. The surgical group included 34 patients who did not respond to pharmacologic intervention and eventually required ligation of their PDA as well as 35 patients who underwent direct ligation because of contraindications to the use of oral ibuprofen. The overall outcomes for the primary and secondary ligation groups were compared. The outcome during hospitalization showed no statistically significant difference in terms of morbidity and mortality between the two groups. The group that had primary ligation for PDA experienced more complications associated with premature birth such as lower gestational age and birth weight. The two groups did not differ significantly in terms of overall outcomes.

## Introduction

Persistent hemodynamically significant ductus arteriosus (HSDA) is considered to be closed, although routine treatment to close a persistently patent ductus arteriosus (PDA) in premature infants is no longer recommended [2, 10]. However, HSDA leads to clinical complications such as pulmonary hemorrhage, bronchopulmonary dysplasia (BPD), necrotizing enterocolitis (NEC), renal impairment, intraventricular hemorrhage (IVH), periventricular leukomalacia, cerebral palsy, and death [2].

Moderate to large left-to-right PDA shunts alter pulmonary mechanics and decrease alveolarization, which results in difficulty weaning the patient off a ventilator [10]. NEC and acute renal failure (ARF) are frequently reported postnatal morbidities that result from pulmonary overcirculation, which is caused by the “ductal steal” phenomenon [18, 23].

The benefits of surgical ligation for the brain and kidneys and the resulting positive effects on NEC in HSDA patients have been reported [16, 21]. As a result, the morbidities associated with HSDA require that clinicians promptly close HSDA with either medication or surgical ligation.

Surgical ligation frequently is the treatment of choice for very-low-birth-weight (VLBW) infants vulnerable to the side effects of cyclooxygenase (COX) inhibitors who are unlikely to experience successful closure with medication. Prostaglandin synthetase inhibitors [6] have a 30–40 % failure rate, and COX inhibitors cannot be prescribed for all patients. Therefore, surgical ligation often is performed after medical treatment failure but also can be performed as the first line of treatment.

With increasing awareness of HSDA closure, our center realized that our target population should be refined such that all patients have HSDA to improve the success rate of surgical intervention. At our center, we adapted the HSDA criterion based on the proposed clinical and echocardiography staging system developed by McNamara and Sehgal [14]. After the HSDA diagnosis, early intervention to close PDA helps to reduce the time of HSDA exposure and minimize the complications associated with PDA.

At our center, patients usually are stabilized and can tolerate weaning off the ventilator and advancement of feeding well within 3 days after surgical ligation of PDA. We compared the overall outcomes between VLBW infants who underwent primary ligation and those who had secondary ligation. This study aimed to evaluate the outcomes in the two HSDA groups during their admission periods.

## Materials and Methods

Between 1 November 2010 and 1 September 2012, 178 VLBW infants were admitted to the Neonatal Intensive Care Unit (NICU) of Seoul St. Mary’s Hospital, the Catholic University of Korea. These infants were enrolled prospectively in this study. Clinical information related to both the presence and treatment of PDA as well as treatment outcomes was collected.

The VLBW infants were screened for PDA within the first 4 days of life, and their diagnosis was confirmed with a two-dimensional echocardiogram. Our target for treatment included infants with at least moderate-stage HSDA (stage ≥3) based on the clinical and echocardiographic criteria developed by McNamara and Sehgal [14]. The VLBW infants with complex congenital heart disease or right-to-left or bidirectional PDA shunting were excluded from the study.

Medical failure was defined as consistent HSDA (stage ≥3) after two courses of oral ibuprofen, a large PDA (4 mm), or any contraindications after the first course of oral ibuprofen treatment. The inclusion criteria for this study specified VLBW infants with at least a moderate stage of HSDA (stage ≥3) based on the clinical and echocardiographic criteria developed by McNamara and Sehgal [14] who could not be treated pharmacologically due to various clinical contraindications.

When pharmacologically indicated, oral ibuprofen was prescribed at 10 mg/kg as a base dose, followed by two additional doses of 5 mg/kg on consecutive days [1, 17]. A PDA surgical ligation was routinely performed at the bedside in the NICU, and all ligations were performed by the same experienced pediatric cardiac surgeon using a metal clip through the third or fourth intercostal space after posterolateral thoracotomy. The procedure required a mean time of 51 min. The study was approved by the Ethics Committee of Seoul St. Mary’s Hospital.

### Definitions

By definition, mild apnea involves episodes of oxygen desaturation, bradycardia or apnea fewer than six times a day, and moderate apnea involves hourly episodes of oxygen desaturation, bradycardia, or apnea [14]. BPD is defined as the use of oxygen exceeding 0.21 % at a corrected gestational age of 36 weeks, at discharge to home of infants born before 32 weeks of gestational age, or at a postnatal age of more than 28 days but less than 56 days for infants born after 32 weeks of gestational age.

Necrotizing enterocolitis, using Bell’s classification, is defined as grade 1 or higher. The treatment of NEC consists of feeding restriction and administration of antibiotics. Oliguria is defined as a urine output exceeding 1 ml/kg/h, and ARF is defined as a sustained decrease in urine output with serum creatinine increased to 1.5 mg/dl or more. IVH is defined as current bleeding in the ventricles (grade ≥2) using the classification constructed by Drs. Papile and Levene. Retinopathy of prematurity (ROP) in this study was defined as neovascular tufts found posterior to the ridge and classified higher than stage 3 according to the International Classification of Diseases for ROP.

### Statistical Analysis

Continuous variables were compared using Student’s *t* test and are expressed as means ± standard deviations. Discrete variables were compared using a *χ* ^2^ test or Fisher’s exact test and are expressed as percentages. All the analyses were two-tailed, and clinical significance was defined as a *p* value lower than 0.05.

For potential differences in neonatal morbidities between the two groups, we used a multivariate logistic regression model. Odds ratios (ORs) and 95 % confidence intervals (CI) were calculated using a multivariate statistical model that included the following predictors related to hospital outcomes with a stepwise logistic regression analysis: birth weight (1,000 g) and gestational age (<27 weeks). All the statistical analyses were performed with the Statistical Package for the Social Sciences (SPSS), version 15.0 (SPSS-PC Inc., Chicago, IL, USA).

## Results

### Demographic Data

Ibuprofen treatment was effective for 109 (61 %) of the 178 infants, but the remaining 69 patients (39 %) required surgical ligation. The surgical group of 69 patients consisted of the 34 patients who did not respond to ibuprofen and the 35 patients directly ligated because of contraindications to the use of oral ibuprofen. In the group treated with ibuprofen, 25 infants (71 %) received one course of ibuprofen, and 10 (29 %) were treated with two courses. The 35 infants directly ligated because of contraindications to oral ibuprofen had bleeding tendencies (positive DIC; *n* = 21), evolving severe IVH (grade ≥3; *n* = 7), large PDA (>4 mm; *n* = 3), poor urine output (<1 ml/kg/h; *n* = 2), and ARF (*n* = 2).

Clinical data were compared between the two groups (Table 1). The gestational age (27.3 vs 29.2; *p* < 0.048) and birth weight (1.02 vs 1.19; *p* < 0.037) were significantly lower only in primary surgical group. Males outnumbered females in both groups (0.56 vs 0.52).

**Table 1:** Clinical characteristics of the primary ligation group versus the secondary ligation group

		Primary ligation (*n* = 35)	Secondary ligation (*n* = 34)	*p* value
Gestational age (days)	192.88 ± 21.88		203.24 ± 17.84	0.048^a^
Birth weight (kg)	1.02 ± 0.36		1.19 ± 0.28	0.037^a^
Apgar score at 1 min	3		4	0.074
Apgar score at 5 min	5		6	0.017
Males, *n* (%)	14 (40.0)		18 (53.0)	0.773
RDS, *n* (%)	30 (85.7)		31 (91.2)	0.286
PDA shunt size at initiation (mm)	2.16 ± 1.08		2.04 ± 1.10	0.342
PDA shunt size at ligation (mm)	3.16 ± 1.38		3.24 ± 1.10	0.482
Duration of admission (days)	70.96 ± 44.75		66.19 ± 30.25	0.655
Duration of MV (days)	39.85 ± 33.26		32.35 ± 28.47	0.352
Duration of O_2_ therapy (days)	57.58 ± 44.21		54.21 ± 33.80	0.739

*RDS* respiratory distress syndrome, *PDA* patent ductus arteriosus, *MV* mechanical ventilation, *O*_*2*_ oxygen

^a^Fisher’s exact test

The initial PDA shunt size was approximately 2 mm, but it was larger (~3 mm) at the time of ligation. However, the initial PDA shunt size and the PDA size at the time of ligation did not differ significantly between the two groups. Additionally, the median duration of admission, ventilation, and oxygen did not differ significantly between the two groups. The mean age at the time of ligation was 14.5 days in the primary ligation group and 20.5 days in the secondary ligation group.

### Primary Versus Secondary Ligation

The outcome during hospitalization showed no statistically significant difference in the rates of BPD, NEC (grade ≥1), ARF, IVH (grade ≥2), periventricular leukomalacia, ROP, culture-proven sepsis, or mortality between the two groups (Table 2). No immediate intraoperative complications such as vocal cord paralysis, hemorrhage, air leaks, wound infection [11]^,^ or chylothorax were noted in either group.

**Table 2:** Outcomes compared between the primary ligation and secondary ligation groups

Morbidity	Primary ligation (*n* = 35)	Secondary ligation (*n* = 34)	*p* value
CLD	19 (73.1 %)	25 (73.5 %)	0.969
Severe CLD	12 (41.4 %)	10 (29.4 %)	0.826
NEC (grade ≥1)	8 (30.8 %)	11(32.4 %)	0.896
ARF	14 (53.8 %)	12 (35.3 %)	0.151
IVH (grade ≥2)	19 (73.1 %)	21 (61.8 %)	0.357
ROP	8 (30.8 %)	10 (29.4 %)	0.909
PVL	5 (19.2 %)	5 (14.7 %)	0.641
Sepsis	14 (53.8 %)	15 (44.1 %)	0.455
Death	5 (14 %)	3 (9 %)	0.072

*CLD* chronic lung disease, *NEC* necrotizing enterocolitis, *ARF* acute renal failure, *IVH* intraventricular hemorrhage, *ROP* retinopathy of prematurity, *PVL* periventricular leukomalacia

All the infants were ventilated at the time of ligation. Severe IVH (grades 3 and 4) was diagnosed for seven infants (20 %) in the primary ligation group, compared with five infants (15 %) in the secondary ligation group, but the difference between the two groups was not significant. In addition, the two groups did not differ significantly in terms of increase in ventilation, fraction of inspired oxygen (FiO_2_), or pressor requirement in the 48 h after ligation. Additionally, the findings showed no significant difference in the incidence of postoperative complications. At the time of NICU discharge, the two groups did not differ significantly with regard to rates of mortality, moderate to severe BPD (41 vs 29 %), ROP (31 vs 29 %), or NEC (31 vs 32 %) (Table 2).

### Short-Term Outcomes After Surgical Ligation

The primary reasons for ligation were pulmonary edema or cardiomegaly shown by radiology (*n* = 28, 40 %), an increasing requirement for ventilation (*n* = 25, 36 %), systemic hypotension requiring at least a single cardiotropic agent (*n* = 20, 29 %) that could not be explained by other causes, hourly episodes of oxygen desaturation (*n* = 26, 38 %), and/or mild metabolic acidosis (pH 7.1–7.25 and/or base deficit of −7 to −12) (*n* = 18, 26 %).

The infants born at 24–27 weeks of gestation were more likely to meet the criteria for ligation (92 %) than those born after 27 weeks of gestation (56 %). Of the patients with moderate apnea, 30 (43 %) improved to mild apnea with occasional (<6) episodes of oxygen desaturation or bradycardia [14]. A majority of the patients (93 %) were able to continue enteral feedings and increased their feeding amounts (10–20 ml/kg/day) 3 days after ligation. The oliguria experienced by 50 of the infants (72 %) was resolved by day 3 after ligation. Although the group with primary surgical closure of PDA had more impairments related to immaturity than the group that had secondary surgical closure, no differences in the rates of morbidity or mortality were found based on our logistic regression analysis during hospitalization (Table 3).

## Discussion

In this study, the PDA group with primary surgical ligation experienced more complications related to premature birth, such as gestational age and birth weight. However, the two groups did not differ significantly in overall outcomes.

The use of prostaglandin synthetase inhibitor for PDA has a 30–40 % failure rate [6], and 40 % of the patients are unlikely to respond to indomethacin therapy [4, 13, 20]. Our failure rate for oral ibuprofen was similarly 39 %.

Infants who are more premature and weigh less also have a higher prostaglandin synthetase inhibitor failure rate [19]. The reported complications include pulmonary bleeding, NEC, intestinal perforation, renal failure, and thrombocytopenia, especially in low-birth-weight preterm neonates. Therefore, prostaglandin synthetase inhibitors are used with great caution for VLBW infants and may even worsen already compromised intestinal blood flow in the presence of a hemodynamically significant PDA [26].

Additional studies also have proved that treatment failure is associated with low birth weight (<1,500 g) and severe cardiopulmonary compromise [22, 27]. Mellander et al. [15] found that pharmacologic failure for neonates with cardiopulmonary compromise results in prolonged ventilator support after surgical ligation of their ductus.

Because surgical ligation is an effective and definite procedure associated with low mortality, when indicated, timed ligation without delay of the HSDA exposure period for VLBW infants may be considered. The optimal timing for surgical ligation is debatable. The study by Lee et al. [12] performed in the United Kingdom showed an association between multiple courses of COIs before PDA ligation and an increased incidence of chronic lung disease.

A similar trend in the data associated with delayed ligation raises the possibility that earlier treatment could be advantageous [7]. The patients referred early for ligation had evidence of improved respiratory outcomes [7]. Early surgical closure, compared with late closure, is associated with improved short-term ventilator parameters and pulmonary function, more rapid advancement to full oral feeding, and healthier body growth [24]. Jaillard et al. [8] studied the consequences of delayed surgical closure of PDA in very premature infants and concluded that early surgical closure of the ductus arteriosus (at <3 weeks of life) is associated with a shortened delay in full oral feeding and improved body growth compared with late surgical closure (at >3 weeks of life). The optimal timing of ligation may depend not only on postnatal age (at less than 3 weeks of life) but also on when the HSDA develops and how long the significant ductus arteriosus exposes and hemodynamically affects related organs.

In general, pulmonary overcirculation may lead to considerable morbidities involving CLD, NEC, and renal impairment due to the “ductal steal” phenomenon. A recent study of premature baboons showed altered pulmonary mechanics and arrested alveolarization after 14 days of exposure to moderate-sized ductus [3]. Overcirculation to the lungs in humans also can interfere with the adequate development of premature lungs and could result in microcirculatory lung damage [25].

**Table 3:** Effects on neonatal morbidity and mortality in the primary ligation group versus the secondary ligation group

Morbidity	OR	95 % CI	*p* value
CLD	0.171	0.396–5.141	0.587
NEC (grade ≥1)	1.920	0.525–7.019	0.324
ARF	0.718	0.215–2.404	0.289
IVH (grade ≥2)	1.001	0.269–3.722	0.999
ROP	1.474	0.392–5.544	0.566
PVL	0.972	0.215–4.400	0.970
Sepsis	1.018	0.327–3.167	0.976
Death	0.283	0.075–1.953	0.248

*OR* odds ratio, *CI* confidence interval, *CLD* chronic lung disease, *NEC* necrotizing enterocolitis, *ARF* acute renal failure, *IVH* intraventricular hemorrhage, *ROP* retinopathy of prematurity, *PVL* periventricular leukomalacia

Two small randomized controlled trials performed almost 30 years ago were designed to examine the effects of a persistent symptomatic PDA on neonatal pulmonary morbidity [5, 9]. Both studies found that surgical closure of PDA decreased the need for prolonged ventilator support, whereas significant pulmonary morbidity occurred for patients without PDA ligation when signs of congestive failure developed [5, 9]. Benitz [2] additionally suggested that delayed ductal closure in premature infants is a reflection of an underlying process, such as systemic inflammation, that contributes to various morbidities in addition to the associated large left-to-right ductal shunting. They further mention that a particular class of treatments (intervention to close the PDA) that fail to improve outcomes does not mean that no treatment is useful or necessary or that the PDA can simply be ignored [2].

In conclusion, when HSDA is found in VLBW infants and needs to be closed, timed ligation may be beneficial for reducing pulmonary, renal, and intestinal morbidities. The optimal timing of surgical ligation in the case of HSDA is as early as possible according to the proposed clinical and echocardiography staging system developed by McNamara and Sehgal [14].

Our study demonstrated that primary ligation is safe, without postoperative complications, and that the risks after HSDA exposure, which can cause IVH, NEC, CLD, and increased mortality in VLBW infants, might be reduced. Ligation of PDA also was associated with short-term respiratory and feeding improvements in our study. Careful patient selection with prompt intervention may be needed, and timely ligation for PDA can be an ideal treatment method for HSDA when pharmacologic interventions are unsuccessful.
